# Salivary cystatin S levels in children with early childhood caries in comparison with caries-free children; statistical analysis and machine learning

**DOI:** 10.1186/s12903-021-02016-x

**Published:** 2021-12-18

**Authors:** Maryam Koopaie, Mahsa Salamati, Roshanak Montazeri, Mansour Davoudi, Sajad Kolahdooz

**Affiliations:** 1 Department of Oral Medicine, School of Dentistry, Tehran University of Medical Sciences, Tehran, Iran; 2 Department of Pediatric Dentistry, School of Dentistry, Tehran University of Medical Sciences, North Kargar St, P.O.BOX:14395 -433, 14399-55991 Tehran, Iran; 3 Department of Computer Science and Engineering and IT, School of Electrical and Computer Engineering, Shiraz University, Shiraz, Iran; 4 Universal Scientific Education and Research Network (USERN), Tehran, Iran

**Keywords:** Early childhood caries (ECC), Saliva, Cystatin S, Machine learning

## Abstract

**Background:**

Early childhood caries is the most common infectious disease in childhood, with a high prevalence in developing countries. The assessment of the variables that influence early childhood caries as well as its pathophysiology leads to improved control of this disease. Cystatin S, as one of the salivary proteins, has an essential role in pellicle formation, tooth re-mineralization, and protection. The present study aims to assess salivary cystatin S levels and demographic data in early childhood caries in comparison with caries-free ones using statistical analysis and machine learning methods.

**Methods:**

A cross-sectional, case–control study was undertaken on 20 cases of early childhood caries and 20 caries-free children as a control. Unstimulated whole saliva samples were collected by suction. Cystatin S concentrations in samples were determined using human cystatin S ELISA kit. The checklist was collected from participants about demographic characteristics, oral health status, and dietary habits by interviewing parents. Regression and receiver operating characteristic (ROC) curve analysis were done to evaluate the potential role of cystatin S salivary level and demographic using statistical analysis and machine learning.

**Results:**

The mean value of salivary cystatin S concentration in the early childhood caries group was 191.55 ± 81.90 (ng/ml) and in the caries-free group was 370.06 ± 128.87 (ng/ml). T-test analysis showed a statistically significant difference between early childhood caries and caries-free groups in salivary cystatin S levels (p = 0.032). Investigation of the area under the curve (AUC) and accuracy of the ROC curve revealed that the logistic regression model based on salivary cystatin S levels and birth weight had the most and acceptable potential for discriminating of early childhood caries from caries-free controls. Furthermore, using salivary cystatin S levels enhanced the capability of machine learning methods to differentiate early childhood caries from caries-free controls.

**Conclusion:**

Salivary cystatin S levels in caries-free children were higher than the children with early childhood caries. Results of the present study suggest that considering clinical examination, demographic and socioeconomic factors, along with the salivary cystatin S levels, could be usefull for early diagnosis ofearly childhood caries in high-risk children; furthermore, cystatin S is a protective factor against dental caries.

**Supplementary Information:**

The online version contains supplementary material available at 10.1186/s12903-021-02016-x.

## Background

Dental caries is one of the most common chronic infectious diseases in children, and it is a major health problem in many countries. Early childhood caries (ECC) is defined as the presence of one or more decayed (non-cavitated or cavitated lesions), missing teeth (because of caries), or filled tooth surfaces in any primary tooth in a child under 72 months of age [1, 2]. The negative impacts of ECC on children’s quality of life are numerous. ECC impair nutrition, speaking, occlusion, social behaviors, and the patient’s self-esteem. The prevalence of ECC in preschool children varies across countries; Furthermore, ECC is a common infectious disease in the majority of countries all over the world [3].

Dental caries is a multifactorial disease that results from multiple factors, including immature immune systems, individual characteristics of saliva, cariogenic microorganisms, hyposalivation, enamel hypoplasia, enamel defects, cariogenic diet, night breastfeeding, and oral hygiene care in early childhood [4]. Premature birth, low birth weight, and lack of access to nutritional supplements during pregnancy are other risk factors of ECC [5]. Socioeconomic factors such as parent’s income and educational level, birth order, and dental insurance coverage are the other influential factors on caries risk [6]. Recently salivary components and characteristics have been introduced as one of the crucial factors affecting ECC development [7]. ECC treatment generally does not have a long-term impact on the population of oral *Streptococcus mutans*. Although these treatments help control the disease, caries recurrence is still common around or after repair, with a recurrence rate of approximately 40% reported during the first year [8, 9].

Saliva is a unique body fluid that is easily accessible and contains complex components with great diagnostic value. Saliva collection and storage are simple, non-invasive, and inexpensive. [10]. The salivary immune system is composed of proteins with a significant effect on oral health. Many salivary proteins prevent the adhesion and aggregation of cariogenic bacteria, and some others have a role in defense against microorganisms [11]. Qualitative and quantitative changes in salivary proteome have a significant role in the initiation and intensity of oral disease [12]. Although protein components of saliva are only 30% of the blood components, it is still known as a rich source of biomarkers [7]. Cystatins are a large family of proteins that control and reversibly inhibit the activity of extracellular cysteine proteinases in inflammatory conditions [13]. Cystatin S is a phosphorylated protein that is found in the tear and saliva. It is mainly secreted by the submandibular gland and to a much lesser extent by the parotid and sublingual gland [14]. Unlike other proteins in this group, cystatin S has a lower cysteine proteinase inhibitory function [15].

Cystatin S has four sites for phosphorylation that bond with hydroxyapatite and has an important role in dental pellicle formation and calcium and phosphate equilibrium and cause enamel remineralization. It also prevents enamel demineralization by attachment to the enamel surface. Cystatin S protects the oral mucosa by its antimicrobial and antiviral effects [16, 17].

Artificial intelligence (AI) is a computer's ability to perform reasoning processes that generally associate with intelligence in human beings. Machine learning is one of the most common forms of artificial intelligence that consists of a set of instructions for processing and finding patterns in large data sets to enable decision-making without human intervention [18]. A large quantity of data in the healthcare field, from clinical symptoms to imaging features, requires machine learning algorithms for classification and regression tasks. As the result of applying machine learning algorithms to clinical data, patients and clinicians benefit in many different ways, such as clinical decision support and the development of clinical care guidelines [19].

Considering the role of cystatin S in caries prevention, this study aims to compare the salivary level of cystatin S in ECC patients and caries-free (CF) children.

## Methods

### Ethical statement

This study was approved by the Tehran University of Medical Sciences Ethical Committee (ethical code: IR.TUMS.DENTISTRY.REC.1398.099) and this study was conducted in accordance with the Declaration of Helsinki [20]. After describing the study objectives, written informed consent was obtained from a parent or guardian for participants under 16 years old. The authors confirm that all methods were performed in accordance with the relevant guidelines and regulations.

### Samples

A cross-sectional case–control study was undertaken on 20 cases of ECC and 20 CF children as a control group. The participants enrolled in this study were selected from children referred to the dental clinic of Tehran University of Medical Sciences (Tehran, Iran) for routine oral examinations. The oral health status of each participant was determined by three professional dentists. The inclusion criteria were children aged between 48 and 72 months with one or more decayed (non-cavitated or cavitated lesions), missing teeth (because of caries), or filled tooth surfaces in any primary tooth. CF controls were matched regarding age and gender. Children with no clinical signs of early caries or white spots were considered to be free of caries and their Decayed, Missing, and Filled Teeth (dmft) index was zero.

After oral examination of participants, the researcher completed the checklist by interviewing parents, which collected information about the demographic characteristics of child and parents, dietary intakes [21], birth weight, oral hygiene behaviors, parental education level, medicine intake, and night breastfeeding. Exclusion criteria include children who had chronic systemic diseases or syndromes, influenza or infection of the respiratory system, taking medicine and received antibiotic therapy within three months, fluoride prophylaxis within 1 year, and medical history of congenital diseases, parents refused to participate or refused to sign the informed consent and children who did not agree or cooperate with the participation. Children were assessed using dmft index based on WHO Oral Health Surveys Basic Methods [22], and diagnosis of ECC was done based on the diagnostic criteria of the American Academy of Pediatric Dentistry [23]. Those having a dmft index of zero were considered CF. After surveying and regarding the exclusion criteria, 20 children were finally selected as cases, and 20 of them were considered as control.

### Saliva collection

Unstimulated whole saliva samples by the suction method were collected. Before sampling, the children were in a rest position and did not eat anything for 30 min. Saliva sampling was done between 9:00 am to 11:00 am to avoid circadian variations in the case and control group. Complete protease inhibitor cocktail (Roche Diagnostics GmbH, Mannheim, Germany) was added immediately after the completion of saliva collection. Saliva samples were centrifuged at 10,000 × g for 15 min at 4 °C; the supernatant was obtained and stored in − 80 °C.

### Determination of salivary cystatin S levels

Cystatin S concentrations were determined using human cystatin enzyme-linked immunosorbent assay (ELISA) kit (ZellBio GmbH, Ulm, Germany). The samples were thawed at 25 °C and assayed in accordance with the manufacturer’s instructions. The absorbance of samples at 450 nm was measured using Hyperion ELISA microplate reader. The concentrations of cystatin S were determined by spectrometer software based on standard curves.

### Statistical analysis

Statistical analysis was performed with SPSS software (version 22; SPSS Inc., Chicago, IL, USA) and GraphPad Prism 8.2.1 for Windows (GraphPad Software, San Diego, California). In order to assess the relationship between dental caries and age with cystatin S salivary level, T-test, and Levenes' test were used. Mean value and standard deviation of cystatin S levels were reported. Regression analyses were performed for cystatin S level with the backward stepwise method. The contribution of each variable (including ECC, demographic and clinical characteristics, and nutrition habits) was expressed by the p-value (p) and standardized coefficients beta (β). The receiver operating characteristic (ROC) curve analysis was done to evaluate the potential role of cystatin S salivary level and combination of cystatin S salivary level with weight of birth for early diagnosis of ECC. All results are presented as mean ± standard deviation (SD), and p ≤ 0.05 was considered statistically significant.

### Machine learning analysis

We applied multiple supervised machine learning methods to assess the usefulness of cystatin S in addition to demographic and clinical factors, besides nutrition habits in predicting ECC. Supervised learning models are trained using labeled training data, where the model learns about each type of data. After the training phase is completed, the model is evaluated on the test data to predict the type of unlabeled data. In this paper, several supervised learning models such as feed-forward neural networks, XGBoost, Random Forest, and Support vector machine (SVM) are used to generalize our findings.

For implementing a feed-forward neural network (Additional file 1: Figures. S1 and S2), we utilized Python Software Foundation, Version 3.7, and the open-source deep learning package, namely Keras [24], which is a high-level neural network API. The proposed feed-forward neural network model has two hidden layers, and each hidden layer has 32 neurons (Additional file 1: Figures S1, S2). We used the ReLU^1^ activation function for the hidden layers, and for the output layer, the sigmoid activation function was used. We utilized the binary cross-entropy method as the loss function. In order to set the hyper-parameters, we used the fivefold cross-validation method, and the Adam optimizer [25] technique was utilized to update the weights and bias parameters of the neural network layers. Additionally, we utilized the scikit-learn package^2^ to construct the previously discussed supervised learning methods, including XGBoost, Random Forest, and Support vector machine, and ran all classifiers with default parameter settings. To assess the effectiveness of cystatin S levels in predicting ECC, for each supervised method, we construct two models:

- The first model uses the clinical features, patient characteristics, and nutrition habits as the input features for ECC prediction.
- The second model, in addition to the features used in the first model, utilizes cystatin S levels as the input feature to predict ECC.

The ROC curve analysis, in addition to the rate of accuracy, sensitivity, and specificity measures of two constructed models, are provided in the machine learning results section.

## Results

### Patient characteristics

The demographic and clinical characteristics of forty sex- and age-matched subjects from the CF and ECC groups (20 subjects from each group) are depicted in Table 1.

**Table 1:** Participants demographic and clinical characteristics

	**Control (CF) (n = 20)**	**Patients (ECC)** **(n = 20)**
Age of participants, months (mean ± SD)	63.70 ± 8.32	63.25 ± 8.32
Sex		
Male	8 (40%)	11(55%)
Female	12(60%)	9(45%)
Birth order		
First child	8 (40%)	11 (55%)
Second child	8 (40%)	5 (25%)
Third child	3 (15%)	2 (10%)
Forth child	1 (5%)	2(10%)
Father’s education level (based on ISCED 2011 [26])		
Post-secondary non-tertiary education and other lower educational levels (ISCED 4 and lower levels)	1 (5%)	6 (30%)
Short-cycle tertiary education (ISCED 5)	2 (10%)	13 (65%)
Bachelor or equivalent (ISCED 6)	11 (55%)	0 (0.0%)
Master or equivalent (ISCED 7)	6 (30%)	1 (5%)
Mother’s education level (based on ISCED 2011 [26])		
Post-secondary non-tertiary education and other lower educational levels (ISCED 4 and lower levels)	0 (5%)	4 (30%)
Short-cycle tertiary education (ISCED 5)	5 (10%)	15 (65%)
Bachelor or equivalent (ISCED 6)	2 (55%)	0 (0.0%)
Master or equivalent (ISCED 7)	13 (30%)	1 (5%)
Father’s age, years (mean ± SD)	35.10 ± 4.75	38.50 ± 4.74
Mother’s age, years (mean ± SD)	32.70 ± 3.93	33.75 ± 5.02
Diseases of parents		
Diabetes	1 (5%)	2 (10%)
Rheumatism	1 (5%)	1 (5%)
Cardiovascular diseases	0 (0%)	1 (5%)
Dental visits regularly (at least every 6 months)		
Yes	17 (85%)	5 (25%)
No	3 (15%)	15 (75%)
Toothbrush		
Yes	19 (95%)	14 (70%)
No	1 (5%)	6 (30%)
Mouthwash		
Yes	7 (35%)	2 (10%)
No	13 (65%)	18 (90%)
Flossing		
Yes	13 (65%)	3 (15%)
No	7 (35%)	17 (85%)
Using fluoride toothpaste		
Yes	18 (90%)	10 (50%)
No	1 (5%)	4 (20%)
Parental smoking daily		
Yes	6 (30%)	3 (15%)
No	14 (70%)	17 (85%)

In the ECC group, 75% of children did not have regular dental visits, but 85% of children had regular dental visits in the CF group. In the ECC group, 95% of children brush daily while 70% of ECC children brush daily. 65% of CF children had daily dental floss; this value was 15% for ECC group. Just 22.5% of children from both groups used regular mouthwash. 75% of CF children brush twice a day, and this value was 30% for ECC group. 70% of children in the case and control group used fluoride toothpaste, and 12.5% used toothpaste without fluoride. There was no difference in smoking by parents in the case and control groups.

### Nutrition habits

The frequency of eating sweet snacks [21] in the case and control group and frequency of eating dairy products in the case and control group [27] are shown in Table 2. 97.5% of children in the case and control group consumed milk overnight in their infancy period. There was no difference in case and control groups in this regard. There is no statistically significant difference between the case and control groups in using multivitamin and calcium supplements during pregnancy by participants’ mothers.

**Table 2:** Participants nutrition habits characteristics

	Control (n = 20)	Patients (n = 20)
Consumption of sweet snacks *		
Once a day	12 (60%)	9 (45%)
Twice a day	8 (40%)	6 (30%)
Three times a day or more	0 (0%)	5 (25%)
Consumption of sour snacks **		
Once a day	12 (60%)	15 (75%)
Twice a day	8 (40%)	3 (15%)
Three times a day or more	0 (0%)	2 (10%)
Consumption of dairy units per day		
0 times a day (didn’t consume)	0 (0%)	1 (5%)
Once a day	3 (15%)	7 (35%)
Twice a day	9 (45%)	7 (35%)
Three times a day or more	8 (40%)	5 (25%)
Consumption of milk at night during infancy		
Yes	19 (95%)	20 (100%)
No	1 (5%)	0 (0%)
Taking vitamin and calcium supplements by the mother during pregnancy		
Yes	18 (90%)	20 (100%)
No	2 (10%)	0 (0%)

^*^ Sweet snacks were defined as any product containing sugar such as cake, biscuit, cocci, wafer, toffee, chocolate, candy, chewing- gum, ice cream, juice, soda, coca and so on

^**^ Sour snacks were defined as snacks with a sour taste, such as sour fruits, sour juice, fruit rolls, and so on

### Statistical analysis

**Fig. 1:**
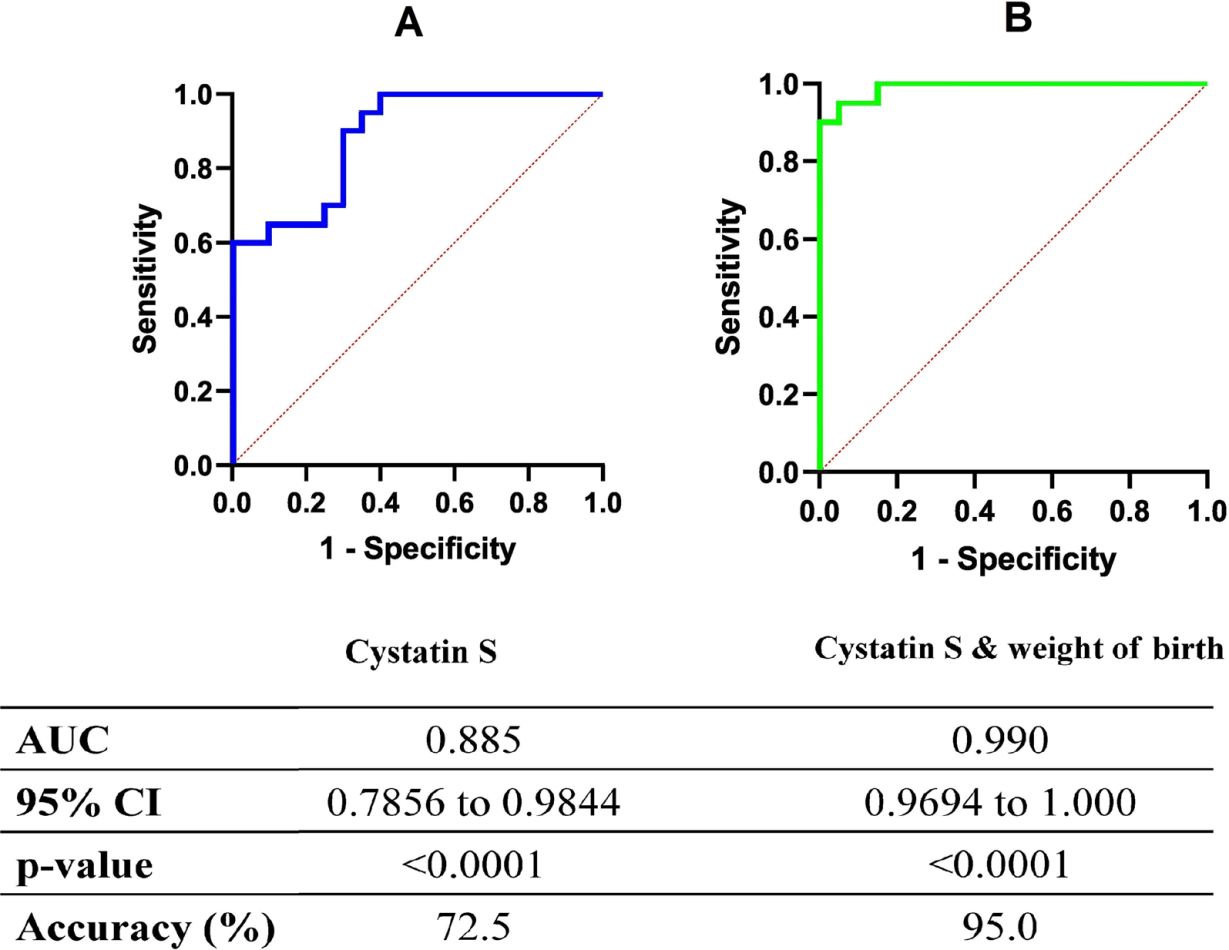
The receiver operating characteristic (ROC) curve analysis by statistical analysis using **a** Cystatin S, **b** Cystatin S & birth weight for discriminating of ECC from CF controls

The mean value of salivary cystatin S concentration in ECC group was 191.55 ± 81.90 (ng/ml) and in CF group was 370.06 ± 128.87 (ng/ml). T-test analysis indicated a statistically significant difference between ECC and CF group in salivary cystatin S levels (p = 0.032). The cut-off value of cystatin S in saliva for differentiation of ECC cases and CF was calculated based on Youden’s index [28, 29]. Considering 306.3 ng/ml as a cut-off value, the sensitivity of salivary cystatin S levels in caries diagnosis was 95%, and its specificity was 65% (Fig. 1-A).

**Fig. 2:**
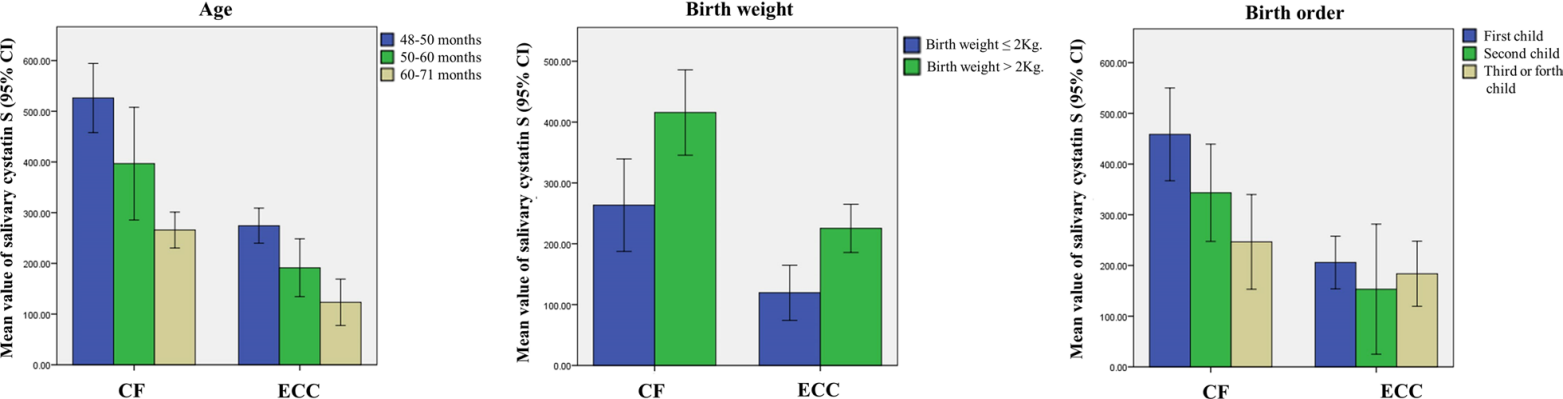
Left) Mean value of salivary cystatin S levels in ECC and CF groups based on the age of the children. Middle) Mean value of salivary cystatin S levels in ECC and CF groups based on the birth weight of the children. Right) Mean value of salivary cystatin S levels in ECC and CF groups based on the birth order of the children

The mean value of salivary cystatin S levels in CF and ECC groups based on the age of children, birth weight, and birth order are shown in Fig. 2.

In order to assess the effect of risk factors with and without ECC on salivary cystatin S levels, backward stepwise regression was performed, and two models were concluded by including variables (criterion: Probability of F-to-remove ≥ 0.100) (Additional file 1). There was a statistically significant relationship between child’s age (p < 0.001, β = − 0.413), birth weight (p = 0.002, β = 0.273), birth order (p = 0.021, β = − 0.152) and ECC (p < 0.001, β = − 0.658) with salivary cystatin S levels in regression model considering risk factors with ECC (Additional file 1).

In addition, there was a statistically significant relationship between the age of children (p < 0.001, β = − 0.533), father education (p = 0.007, β = 0.361) and mother education (p = 0.019, β = 0.308) with salivary cystatin S levels regression modeling of cystatin S level in regression model considering risk factors without ECC. Furthermore, logistic regression was used for modeling and early diagnosis of ECC risk based on the cystatin S and weight of birth (Additional file 2). ROC curve based on the logistic regression modeling for discriminating of ECC from CF control was presented in Fig. 1-B (Additional file 2).

### Machine learning results

In this study, we collected 20 cases of ECC and 20 CF children as a control group. Besides collecting clinical features, patient characteristics, and nutritional habits, we measure cystatin S levels for each case separately to analyze the impact of cystatin S on ECC. For this purpose, we used machine learning and implemented different methods to make our results generalizable. Two machine learning models were constructed to evaluate the effectiveness of cystatin S levels in the differentiation ECC from CF. The first machine learning model includes the mentioned supervised methods that merely used clinical features, patient characteristics, and nutrition habits for ECC early diagnosis. While in the second model, besides clinical features, patient characteristics, and nutrition habits, the cystatin S levels feature was used. Tables 3 and 4 show the rate of accuracy, sensitivity, and specificity measures for the constructed machine learning model, respectively.

**Table 3:** Results of supervised machine learning methods, utilizing all features except cystatin S levels; first machine learning model

All features except cystatin S	Feed-Forward neural network	XGBoost	Random forest	SVM
Accuracy (%)	88.1	85.7	78.5	75
Sensitivity (%)	100	92.8	92.8	92.8
Specificity (%)	71.3	78.5	64.2	57.1

**Table 4:** Results of supervised machine learning methods, utilizing all features with cystatin S levels; second machine learning model

All features	Feed-forward neural network	XGBoost	Random forest	SVM
Accuracy (%)	90.9	89.2	85.7	85.4
Sensitivity (%)	100	93.3	93.3	86.6
Specificity (%)	72.1	84.6	76.9	84.6

After analyzing the results, we observe that:

- Using cystatin S levels in addition to other extracted features improves the accuracy and specificity of all the supervised methods.
- The feed-forward neural network method of the second model, which uses cystatin S levels besides the other features, improves the ECC detection rate significantly and obtains the most accuracy and specificity rate among all the methods in both models.

**Fig. 3:**
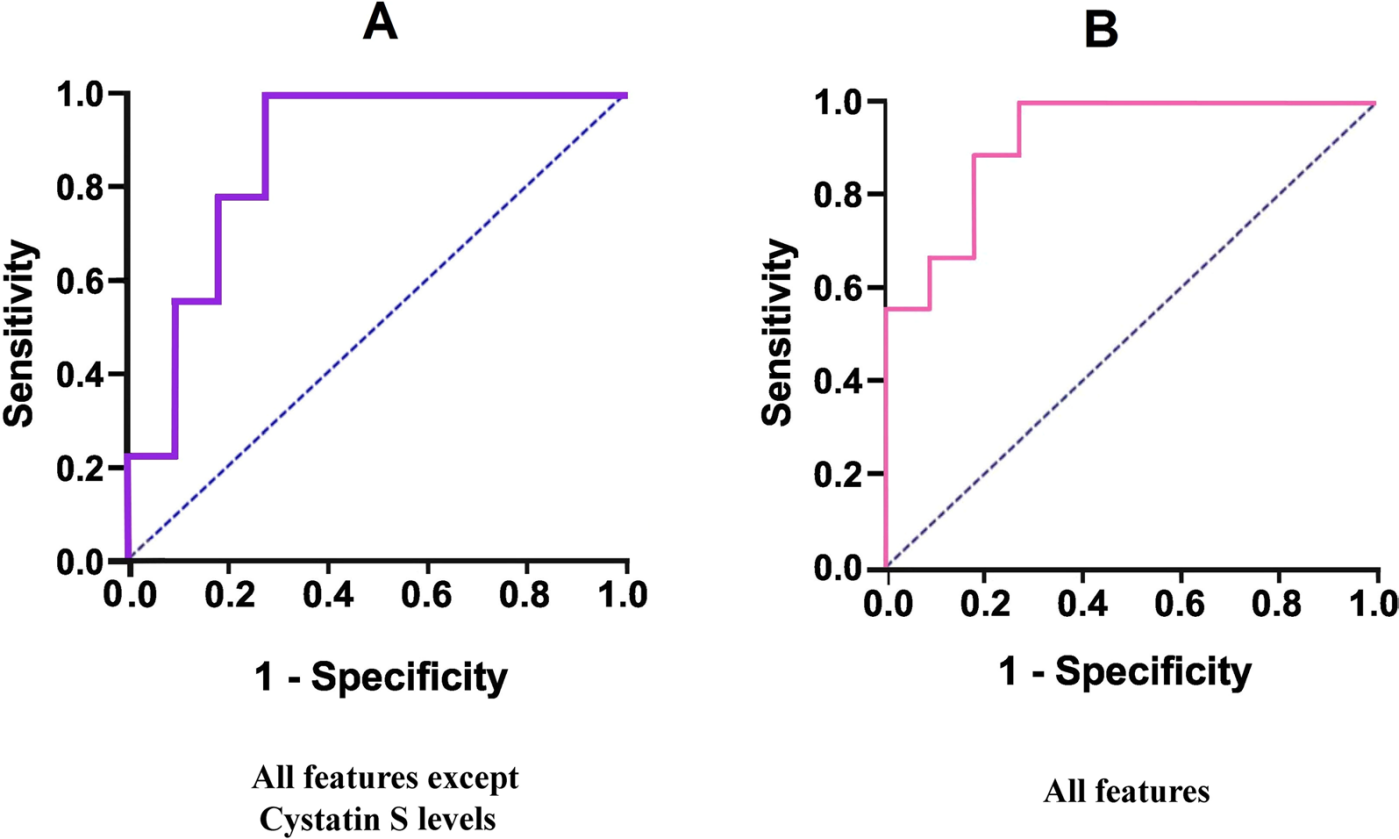
The receiver operating characteristic (ROC) curve by machine learning using C): All features except cystatin S levels, D): All features

ROC curve analysis of the feed-forward neural network method, which uses all features except cystatin S, is shown in Fig. 3-A (Area under the ROC curve (AUC) = 0.87). In addition, ROC curve analysis of the feed-forward neural network method, which utilized all features (including cystatin S levels, demographic and clinical characteristics, and nutrition habits), reveals that the AUC = 0.93 (Fig. 3-B).

**Fig. 4:**
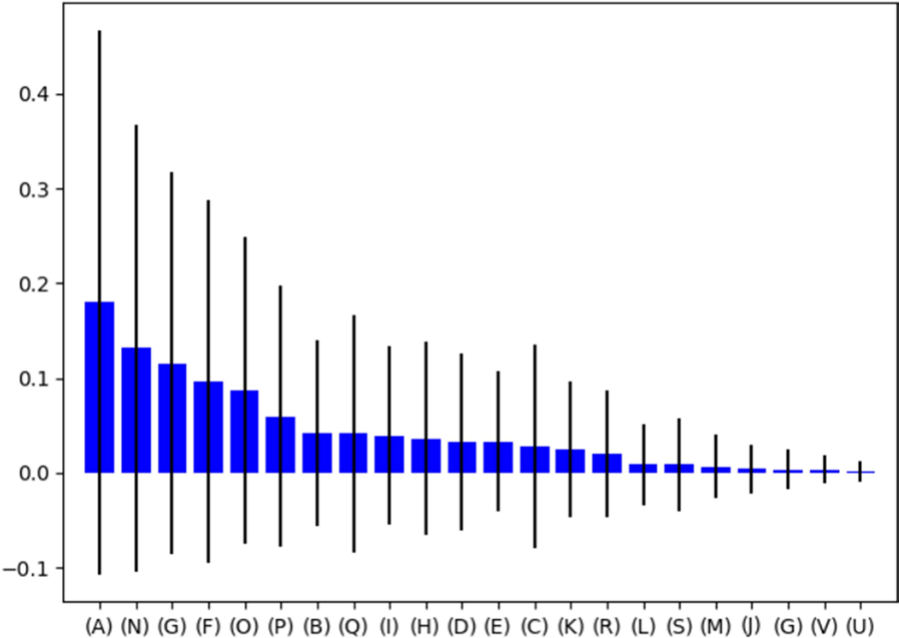
Feature importance score of extracted features in the incidence of ECC using random forest classifier. Features description: A): Cystatin S levels B): Age C): Gender D): Weight of birth E): Birth order F): Father’s education level G): Mother’s education level H): Father’s age I): Mother’s age J): Using Toothbrush K): Flossing L): Using Mouthwash M): Systemic diseases of parents N): Dental visits regularly O): Oral health instrument using P): Number of teeth brushing daily Q): Consumption of sweet snacks R): Consumption of sour snacks S): Consumption of dairy units per day T): Consumption of milk at night during infancy U): Taking vitamin and calcium supplements by the mother during pregnancy V): Parental smoking daily

We also investigated the importance of extracted features in the Random Forest classifier (Fig. 4). Gini impurity [30] was utilized as a feature importance score. Figure 4 demonstrates the feature importance score of extracted features. The “cystatin S levels” were obtained the highest score and second-highest score belong to the “dental visit regularly”, and the third rank belongs to “mother’s education level”.

## Discussion

ECC is premature dental caries in deciduous teeth that mainly affects smooth surfaces and is one of the most common chronic infectious diseases in children [31]. ECC is a preventable disease, and its early diagnosis is critical [32]. Besides diagnosed ECC risk factors, changing salivary composition is one of the risk factors with significant importance in early disease diagnosis [33]. Studies have shown that some salivary compounds have different levels of biological activity in active caries individuals in comparison with CF ones. However, there are limited studies on the difference between salivary protein components in ECC and CF children. Cystatin S is a cystatin protease inhibitor, which is mainly present in submandibular saliva. The phosphorylated form of cystatin S has a significant role in the regulation of salivary calcium levels [34, 35]. Our study assessed salivary cystatin S levels by ELISA revealed that mean value of salivary cystatin S levels was significantly higher in CF children than ECC group (p < 0.005). Furthermore, the sensitivity of cystatin S in caries diagnosis was 95%, and its specificity was 65%. Considering the AUC [36, 37], it could be concluded that cystatin S may be a proper salivary biomarker for early diagnosis of ECC risk.

These results are in line with Vitrino et al. study. They assessed salivary proteins involved in dental pellicle formation. They concluded that the level of cystatin S in dental pellicle in CF individuals was higher than dental caries ones. There was a direct relationship between levels of salivary cystatin S and lower values of dmft [38]. Wang et al., in a study with the aim of salivary proteomics investigation in children with and without caries in the age range of 10–12 years, concluded that the levels of salivary cystatin S in the group without caries is significantly higher than the group with high dental caries [39]. A study by Siqueira et al., compares the protein composition of dental pellicle in patients with and without dental caries. They revealed that cystatin S level in CF group was higher than the dental caries group [40].

Odanaka et al. revealed that cystatin S in dental pellicle originated from saliva [41]. Age is one of the important factors that cause significant changes in saliva composition and plays an important role in determining the proteins of healthy and cariogenic saliva. Cabras et al. stated that the level of salivary cystatin S changes at different ages and their results indicated that the salivary cystatin level increases with aging [42]. Assessment of salivary proteome in the first 48 months of life revealed that cystatin isn’t present in saliva in the first 6 months. It can be concluded that salivary cystatin concentration is age-related and an increase in age and physiologic condition have a great impact on it [43].

The level of cystatin S in the elderly differs from childhood. Preza et al., in a comparison between elderly patients with root caries and elderly patients without root caries as the control group, concluded that the level of parotid cystatin S in elderly patients with root caries was significantly higher than the control group [44]. These results are contrary to the results of our study, which can be attributed to the role of age on the concentration of salivary cystatin S. Regarding the relatively higher expression of salivary cystatin S in CF group, we anticipate a significant relationship between expression of salivary cystatin S and caries risk factors. Considering the high level of cystatin S in CF children, a statistically significant relationship between cystatin S and caries risk factors is anticipated. Julihn suggested that birth order is associated with caries increment in young children [45]. Compared with first-born children, the highest risk of caries increment occurred in fifth- or later-born [45–47]. In our study, there was a statistically significant inverse relationship between expression of salivary cystatin S and birth order. The level of salivary cystatin S in first-born children of the family was higher. These results are consistent with the study by Julihn, which stated that second-, third-,fourth-, and fifth- or later-born children have a significantly increased risk of developing new caries lesions between age 3 to 7 years compared with first-born children [45]. Increasing caries risk by birth order may be attributed to lower attention of parents to oral hygiene habits of children later born. In addition, according to our results, the level of cystatin S in the ECC group is lower than the control group. It could be concluded that by increasing birth order, salivary cystatin s levels decreased.

Another risk factor of dental caries is low birth weight. Some studies demonstrated a significant relationship between dental caries and preterm low birth weight status. Bernabé et al. concluded that low birth weight and maternal smoking cause an increase in caries rate. The results were in line with ours. Nicolau et al. in a study concluded that low birth weight children, which were second-born child in the family, had a higher dmft index [48]. Hallett et al. in their study reported that prevalence of ECC in fourth-born children of the family was significantly higher (p = 0.001) [49].

These differences between siblings can be for a variety of reasons, including the time parents spend with children or changes in the family as a result of the presence of children of different ages. The first child usually gets all the attention of the parents, at least in the first period of life. Although younger children can benefit from the education of their older siblings, this is a mutual benefit, and it has even been said that older children benefit more from teaching their younger siblings. It is also not hard to imagine that many things that children learn from their older siblings may have a negative effect [50].

Birth problems, such as low birth weight, can lead to decreased immune function or enamel hypoplasia and early establishment of cariogenic bacteria in the oral cavity, which increases the risk of dental caries [44]. In our study there was a statistically significant relationship between birth weight and salivary levels of cystatin S which was similar to Rajshekar et al.’s study. They concluded that prevalence of dental caries has a significant relationship with birth weight and low birth weight children had higher dental caries than normal weight children [51].

Bernabé et al. also found that maternal weight and maternal smoking were associated with changes in dmft. Children with low birth weight and smoking mothers showed a higher rate of caries than children with normal birth weight and non-smoking mothers [52]. These results were in line with ours but Athamneh et al. assessed 30–48 months children and concluded that there isn’t a significant relationship between birth weight and dental caries [53].

Some studies have shown that the children of parents with higher education have better oral health. A statistically significant positive correlation between salivary cystatin S levels and parent education was found in ours. Highly educated families have a better economic situation, which leads to more benefits from health services and improving their children's oral health.

Another aspect of the impact of parental education is increasing parental awareness of oral hygiene and caries prevention [54]. The present study did not show a significant relationship between salivary cystatin S levels and oral health and nutritional variables. Based on the results of other studies, frequency of sugary snacks and sugary drink intake significantly affect dental caries in children. Also, there was a higher caries risk in children who often snacked before sleep than those who never snacked before sleep. In this study, the frequency of eating sweet snacks in ECC group was slightly higher than control group but this difference was not statistically significant that may be attributed to small sample size. Regarding oral hygiene habits frequency of toothbrushes and dental flossing in ECC group was lower than control. These results are in line with the other studies, which state that brush at an earlier age and parent-assisted brushing can often reduce the risk of caries [55]. Given that these variables are all part of the risk factors for dental caries and various studies have found a significant relationship between them and dental caries, the result of our study can be due to parents' dishonesty in responding to questions about oral health and nutritional habits because parents tend to show their child's health and eating habits better than they already are.

In addition to studying demographic information, the relationship between demographic data and oral habits with the salivary cystatin S levels was investigated in ECC children in comparison with CF cones. Applying the information mentioned above to a set of machine learning methods confirmed our achieved findings. Using machine learning methods in oral healthcare improves dentist checkup skills and introduces novel and complex cause-and-effect relationships, which are not easily possible by examining and receiving a patient’s history.

Machine learning methods do not cause easily identify crucial factors of diagnosing ECC levels but help us develop computer algorithms that can consider a set of variables and their complicated relationships. Although these complex relationships are challenging for human beings, with the help of machine learning methods, they are comprehensible. By taking advantage of machine learning in clinical issues, many useful facilities in public health are provided. Machine learning can be used as a screening tool in dentistry.

This study was a step towards better understanding the early diagnosis of ECC. One limitation of this study is the sample size. Since this study was performed during the Covid-19 pandemic, we had to reduce the sample size to the minimum acceptable number based on the the amount determined using the sample size equation. There were other limitations, including the use of self-reporting measures for all variables. Although the questioner provided sufficient and convincing explanations to the parents, there was a mistake that the parents were trying to make the child's living conditions better or different from the real ones. Considering the presented limitations, caution is necessary for interpreting this analysis and generalizing it to other groups of children. However, it is suggested to provide more comprehensive models by increasing the sample size and following up in different salivary protein markers and demographic data periods.

## Conclusion

In sum, this study showed that cystatin S protein levels were significantly lower in children with early childhood caries than in CF ones. This study confirms the association between cystatin S protein and dental caries in children aged 48–71 months. In addition, a significant relationship was found with some risk factors of ECC and salivary cystatin S levels by statistical analysis and machine learning. Cystatin S is involved in the process of enamel remineralization and dental pellicle formation and plays a protective role in tooth structure. Therefore, reducing this protective protein in saliva in people with risk factors for caries makes teeth prone to decay.. Considering clinical examination, demographic and socioeconomic factors, along with the salivary cystatin S levels, could be useful for early diagnosis of early childhood caries.

## Supplementary Information

**Additional file 1.** Regression analyses (backward stepwise method) for modeling and early diagnosis of ECC risk based on risk factors.

**Additional file 2.** The receiver operating characteristic (ROC) curve analysis by logistic regression modeling using cystatin S & birth weight for discriminating of ECC from CF controls.

## Data Availability

The datasets used and/or analyzed during the current study are available from the corresponding author on reasonable request. Machine learning code that was used is accessible via the following address: https://codeberg.org/mansur/Salivary_cystatin_S_in_early_childhood_caries.

## Abbreviations

AUC: Area under the ROC curve
AI: Artificial intelligence
CF: Caries-free
Dmft: Decayed, Missing, and Filled Teeth
ECC: Early childhood caries
ELISA: Enzyme-linked immunosorbent assay
ROC: Receiver operating characteristic
SD: Standard deviation
SVM: Support vector machine
WHO: World Health Organization

## Footnotes

1
Rectified linear unit.

2 https://scikit-learn.org/.
