# Supplementary material for: Salivary cystatin S levels in children with early childhood caries in comparison with caries-free children; statistical analysis and machine learning
Source: BMC Oral Health. 2021 Dec 18;21:650. doi: 10.1186/s12903-021-02016-x (PMC8683819; doi:10.1186/s12903-021-02016-x)
Supplement: Supplementary file 2 — Additional file 2. The receiver operating characteristic (ROC) curve analysis by logistic regression modeling using cystatin S & birth weight for discriminating of ECC from CF controls. [file 12903_2021_2016_MOESM2_ESM.docx]

| Table Analyzed | Titanic Data Set |  |  |  |
| --- | --- | --- | --- | --- |
| Dependent variable | ECCCF |  |  |  |
| Regression type | Logistic regression |  |  |  |
|  |  |  |  |  |
| Model |  |  |  |  |
| Parameter estimates | Variable | Estimate | Standard error | 95% CI (profile likelihood) |
| β0 | Intercept | -24.66 | 13.94 | -67.74 to -5.800 |
| β1 | Cystatins levels | -0.1241 | 0.06287 | -0.3161 to -0.04457 |
| β2 | Weight | 18.01 | 9.337 | 6.200 to 47.31 |
|  |  |  |  |  |
| Odds ratios | Variable | Estimate | 95% CI (profile likelihood) |  |
| β0 | Intercept | 1.952e-011 | 3.815e-030 to 0.003028 |  |
| β1 | Cystatins levels | 0.8833 | 0.7290 to 0.9564 |  |
| β2 | Weight | 66050268 | 492.9 to 3.535e+020 |  |
|  |  |  |  |  |
| Model diagnostics | Degrees of Freedom | AICc |  |  |
| Intercept-only model | 39 | 57.56 |  |  |
| Selected model | 37 | 16.37 |  |  |
|  |  |  |  |  |
| Area under the ROC curve |  |  |  |  |
| Area | 0.9900 |  |  |  |
| Std. Error | 0.01050 |  |  |  |
| 95% confidence interval | 0.9694 to 1.000 |  |  |  |
| P value | <0.0001 |  |  |  |
|  |  |  |  |  |
| Classification table | Predicted 0 | Predicted 1 | Total | % Correctly classified |
| Observed 0 | 19 | 1 | 20 | 95.00 |
| Observed 1 | 1 | 19 | 20 | 95.00 |
| Total | 20 | 20 | 40 | 95.00 |
|  |  |  |  |  |
| Negative predictive power (%) | 95.00 |  |  |  |
| Positive predictive power (%) | 95.00 |  |  |  |
|  |  |  |  |  |
| Classification cutoff | 0.5 |  |  |  |
|  |  |  |  |  |
| Pseudo R squared |  |  |  |  |
| Tjur's R squared | 0.8419 |  |  |  |
|  |  |  |  |  |
| Hypothesis tests | Statistic | P value | Null hypothesis | Reject Null Hypothesis? |
| Hosmer-Lemeshow | 0.1554 | >0.9999 | Selected model is correct | No |
|  |  |  |  |  |


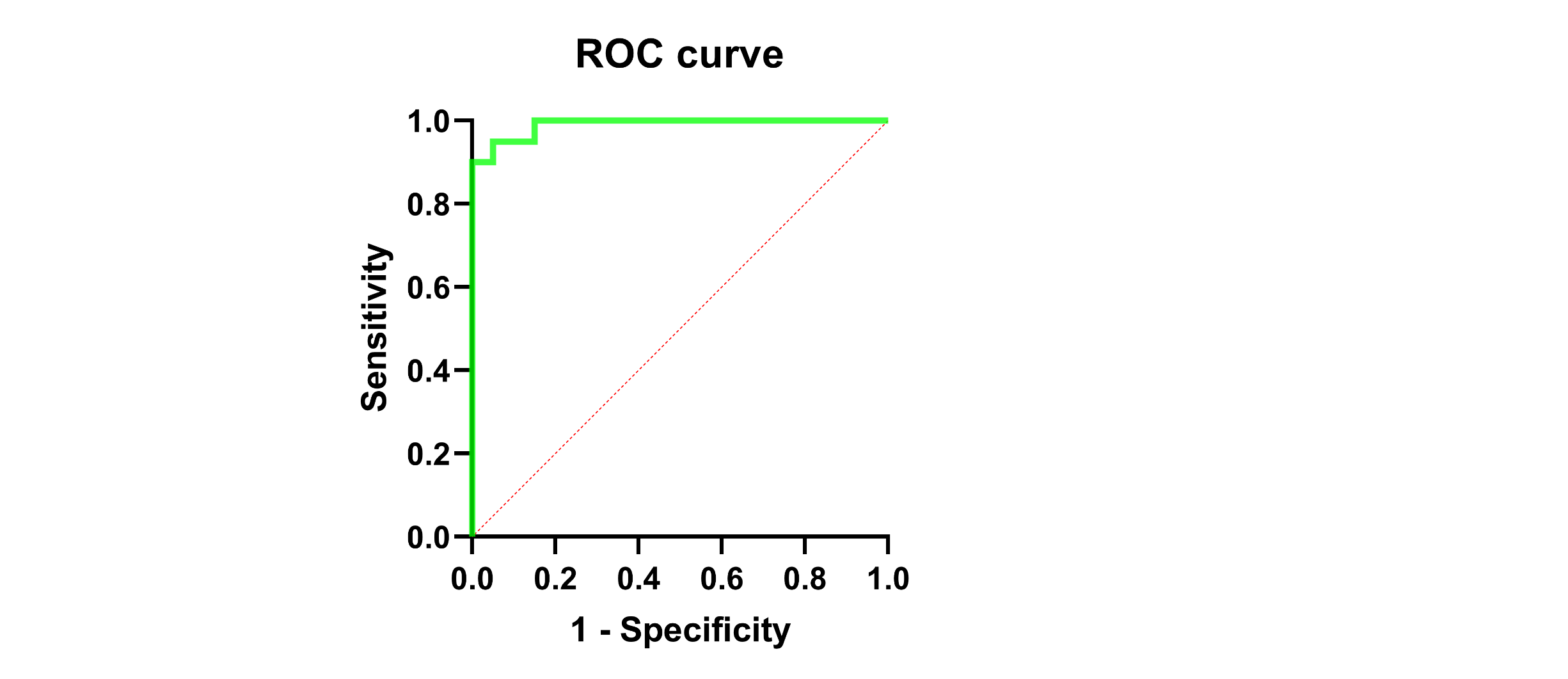


The receiver operating characteristic (ROC) curve analysis by logistic regression modeling using cystatin S & birth weight for discriminating of ECC from CF controls.
